# Functional and Cognitive Impairment in Patients with Relapsing–Remitting Multiple Sclerosis: Cognitive Tests and Plasma Neurofilament Light Chain Levels

**DOI:** 10.3390/medicina61010070

**Published:** 2025-01-03

**Authors:** Elina Polunosika, Joel Simren, Arta Akmene, Nikita Klimovskis, Kaj Blennow, Daina Pastare, Henrik Zetterberg, Renars Erts, Guntis Karelis

**Affiliations:** 1 Department of Neurology and Neurosurgery, Riga East University Hospital, LV-1038 Riga, Latvia; akmene.arta@gmail.com (A.A.); daina.pastare@gmail.com (D.P.); guntis.karelis@gmail.com (G.K.); 2 Department of Neurology and Neurosurgery, Riga Stradinš University, LV-1007 Riga, Latvia; 3 Department of Psychiatry and Neurochemistry, Institute of Neuroscience and Physiology, Sahlgrenska Academy, University of Gothenburg, 40530 Molndal, Sweden; joel.simren@gu.se (J.S.); kaj.blennow@neuro.gu.se (K.B.); henrik.zetterberg@clinchem.gu.se (H.Z.); 4 Clinical Neurochemistry Laboratory, Sahlgrenska University Hospital, 41345 Molndal, Sweden; 5 Faculty of Medicine, Riga Stradinš University, LV-1007 Riga, Latvia; nklimovsky@rsu.edu.lv; 6 Department of Neurodegenerative Disease, UCL Institute of Neurology, London WC1N 3BG, UK; 7 UK Dementia Research Institute, UCL, London WC1E 6BT, UK; 8 Hong Kong Center for Neurodegenerative Diseases, Clear Water Bay, Hong Kong, China; 9 Wisconsin Alzheimer’s Disease Research Center, School of Medicine and Public Health, University of Wisconsin-Madison, Madison, WI 53792, USA; 10 Faculty of Medicine and Life Sciences, University of Latvia, LV-1050 Riga, Latvia; renars.erts@gmail.com; 11 Department of Infectology, Riga Stradinš University, LV-1007 Riga, Latvia

**Keywords:** multiple sclerosis, neurofilament, SDMT, EDSS, BVMT-R, pNfL, 9-HPT

## Abstract

*Background and Objectives:* Multiple sclerosis (MS) is a chronic inflammatory, autoimmune, and neurodegenerative disease of the central nervous system. The disease can manifest and progress with both physical and cognitive symptoms, affecting the patient’s daily activities. The aim of our study was to investigate the correlation between functional status, cognitive functions, and neurofilament light chain levels in plasma in MS patients. *Materials and Methods:* In a cross-sectional study, MS patients with a relapsing–remitting course (according to McDonald’s criteria, 2017) (*n* = 42) from Riga East University Hospital and a control group (*n* = 42) were included. In the MS group, the functional status was determined using the Expanded Disability Status Scale (EDSS), and neurofilament light chain levels in plasma (pNfL) were detected using single molecule array (Simoa) technology. The symbol digit modalities test (SDMT), brief visuospatial memory test—revised (BVMT-R), and the nine-hole peg test (9-HPT) were performed on the MS and control groups, dividing the groups by education level. *Results:* On the SDMT spreading speed, the MS group performed worse than the control group. The median score for the control group was 94.0, and for the MS group, it was 81.3. Slower performance on the SDMT also correlated with a higher EDSS in the MS group. Cognitive processing speed and memory were better in the control group and among individuals with higher education in both groups. For the BVMT-R, we found no difference between the two groups; both groups were able to learn the task equally well, but we found a weak correlation between age and learning in both groups, which could be related to the normal aging process. Execution reaction speed on the 9-HPT with the dominant hand was slower in the MS group (24.1 s) than in the control group (19.4 s). In the MS group, we observed a trend between SDMT performance and pNfL levels: higher pNfL levels were found in individuals who performed more slowly on the SDMT. *Conclusions:* Cognitive and fine motor dysfunction correlates with neurological impairment and plasma neurofilament light chain levels in MS patients.

## 1. Introduction

Multiple sclerosis (MS) is a chronic, autoimmune, inflammatory, neurodegenerative, and demyelinating disease of the central nervous system that primarily affects young, working-age individuals. MS is characterized by an unpredictable course with a wide range of neurological symptoms. There are three main types of MS: relapsing–remitting MS (RRMS), secondary progressive MS (SPMS), and primary progressive MS (PPMS). The classification of MS depends on clinical and radiological activity and disease progression. In all forms of MS, physical disability increases over the course of the disease.

The average age at diagnosis for MS is 32 years [1]. This is typically a time when individuals want to start a family, build a career, and pursue other important life goals. Worldwide, approximately 2.9 million people are living with this diagnosis, with women being affected three times more often than men [1]. About 85% of MS patients are diagnosed with RRMS. MS can manifest with visual impairment, movement and coordination difficulties, lower urinary tract dysfunction, and cognitive impairment. Cognitive impairments affect 40–65% of MS patients [2]. The most commonly affected cognitive domains are information processing speed, complex attention, working memory, visual–spatial abilities, and executive function [3]. These are recognized as frequent consequences that affect the daily lives of individuals with multiple sclerosis, significantly impacting their quality of life and professional activities [4].

Neurofilaments are intracellular cytoskeletal proteins that are highly specific for neurons. There are several subtypes of neurofilaments: heavy chain (NfH), medium chain (NfM), light chain (NfL), α-internexin, and peripherin [5,6]. Neurofilaments are present in neurons of both central and peripheral nervous systems and they are specific markers for axonal damage and central and peripheral nervous system diseases. Neurofilaments can be detected in cerebrospinal fluid and blood. Among the subunits, NfL is currently the most widely studied as a biomarker in neurologic disorders [5]. NfL represents a tissue-specific, objective, and quantitative measure of recent (in the case of serum NfL within the past 3 months) neuronal loss, offering a marker of real-time disease activity [5]. In people with MS, changes in NfL levels from baseline in blood have been demonstrated to be meaningful indicators of the disease worsening and predictors of short- and long-term disease prognosis [7,8,9,10,11].

Functional impairments and decline in cognitive functions can be assessed using appropriate scales and functional tests. This evaluation is conducted for newly diagnosed patients and patients in dynamic monitoring as the disease progresses, regresses, or remains stable.

## 2. Study Objectives

The purpose of this study is to investigate the correlation between functional status, cognitive functions, and neurofilament light chain levels in plasma in MS patients.

## 3. Materials and Methods

### 3.1. Participant Selection

In this cross-sectional study, patient recruitment took place at the Riga East University Hospital; MS patients with relapsing–remitting courses with immunomodulating therapy were selected whose disease duration was less than 20 years and where there had been no relapses in the past three months. But, in the present study, we did not include what specific treatment the patient received. The participants in the control group were randomized and volunteered for this study according to the average age of the MS group. During the visit, neurological status and the EDSS were evaluated, a blood sample was taken and centrifuged, and the brief visuospatial memory test—revised, the symbol digit modalities test, and the nine-hole peg test were administered.

Inclusion criteria for the MS group included a confirmed diagnosis of RRMS based on the McDonald criteria (2017) [12], participants aged between 18 and 62 years, a disease duration of no longer than 20 years, and the ability to understand and perform the required tasks.

Exclusion criteria for the MS group included patients with PPMS or SPMS and clinically isolated syndrome, patients with a disease duration of more than 20 years, and patients who were unable to understand and perform the required tasks. Additional exclusion criteria were a history of stroke, atrial fibrillation, myocardial infarction, chronic kidney disease, pregnancy, diabetes, and individuals who were obese (BMI > or = 30).

Inclusion criteria for the control group included patients with no diagnosis of MS and participants aged between 18 and 62 years with the ability to understand and perform the required tasks.

Exclusion criteria for the control group included a history of severe head trauma, head surgeries, excessive use of alcohol and other intoxicating substances or diagnosed conditions related to cognitive and/or motor deficits, a history of stroke, atrial fibrillation, myocardial infarction, chronic kidney disease, pregnancy, diabetes, and individuals who were obese (BMI > or = 30).

### 3.2. Neurological Assessment of Patients

Data on the duration of illness in years were obtained and recorded from the patients’ medical records. During the visit, a functional evaluation was performed using the Expanded Disability Status Scale (EDSS). The EDSS is a method of quantifying disability in MS patients and monitoring disease progression over time. The EDSS includes measures of eight functional systems by neurologists: visual, pyramidal, cerebellar, brainstem, sensory, bowel and bladder, cerebral, and ambulation. The EDSS ranges from 0 to 10, with increments of 0.5 units. EDSS scores from 0 to 5.5 refer to MS patients who have mild neurological impairment and are able to walk without assistance; patients with serious functional impairment who require assistance with walking and daily activities are scored 6.0–8.5; and patients with scores greater than 9.0 are bedridden, and death due to MS is scored at 10.

### 3.3. Brief Visuospatial Memory Test—Revised

During the visit, the brief visuospatial memory test—revised (BVMT-R) was administered as part of a comprehensive neuropsychological battery as a screening tool and as a repeatable measure to document changes over time. The BVMT-R is widely used as a visual modality assessment tool [13] in studies and clinical practice. This test is used to evaluate visual–spatial memory and memory abilities. The kit includes six task cards with geom [etric figures. The respondent selects one card and is asked to draw the six geometric figures from memory three times in a row. Then, each figure is evaluated based on its accuracy and location according to the scoring criteria. Total recall across all three trials is considered to be the most sensitive indicator of visual–spatial recall abilities [13].

### 3.4. Symbol Digit Modalities Test

The symbol digit modalities test (SDMT) is the most sensitive tool for detecting cognitive function impairments in MS and other conditions. The SDMT measures sustained attention, visual scanning, processing speed, and motor speed. The test can be completed in less than 5 min, making it a simple, quick, economical, and effective tool to dynamically monitor patients and detect cognitive impairments in a timely manner for intensive patient daily visits [14]. This test can be applied to a wide range of patients. It can be administered both in written and oral forms, making it suitable for testing patients with motor or speech impairments. The use of digits in the test results also eliminates language barriers [14]. The test consists of symbols and digits; nine abstract symbols are paired with a number ranging from 1 to 9. The participants are required to write down the number corresponding to each symbol as fast as possible within 90 s. In the written version of the test, the person fills in the numbers that correspond to the symbols, whereas in the oral version, the examiner records the numbers spoken by the participant. In the present study, we conducted a one-time test using the SDMT in its written version.

### 3.5. Nine-Hole Peg Test

Participants were assessed using the Rolyan nine-hole peg test (9-HPT) to measure manual dexterity and fine motor skills. The 9-HPT apparatus consisted of a rectangular board with nine holes equidistantly spaced. Nine cylindrical pegs were used in the assessment. Prior to the test, the participants were given standardized instructions and one practice trial. The test was administered separately for the dominant and nondominant hand, with the order counterbalanced across participants. The participants were instructed to take the pegs from a container, one by one, place them into the holes on the board as quickly as possible, and then replace the pegs back into the container as quickly as possible. The timer was started when the participant picked up the first peg and stopped when the last peg was correctly placed. The test unit is in seconds.

### 3.6. Neurofilament Light Chain Levels in the Plasma

In the MS group, plasma samples during the visit were collected in 3 ml EDTA-K2 tubes and centrifuged at 2200× *g* for 10 min at +20 °C. Polypropylene Pasteur pipettes were used to store plasma samples in 1.0 mL labeled aliquots at −80 °C. Plasma neurofilament light (pNfL) concentrations were measured using single molecule array (Simoa) technology on an HD-X analyzer using the commercially available NF-Light assay (Quanterix, Billerica, MA, USA) and the standard protocol [9]; the pNfL value was given in pg/mL.

There are numerous factors that can influence the level of the NfL level in serum, such as demographics, lifestyle, comorbidity, exercise, and body mass index. NfL levels measured in the morning are more than 10% higher than those measured in the evening. Caution should be taken when interpreting NfL levels when disease treatment-induced neurological complications can potentially impact NfL levels. Serum NfL levels can be decreased in patients with high levels of immunoglobulin G (IgG). Higher concentrations of NfL may be found in people with a history of stroke, atrial fibrillation, myocardial infarction, chronic kidney disease, pregnancy, and diabetes. A lower concentration of NfL may be found in individuals who are obese (BMI > or =30) [15,16,17,18,19]. In the present study, appropriate patients according to inclusion and exclusion criteria were selected, and blood samples were collected in the morning.

### 3.7. Data Statistical Analysis

The data were analyzed using R version 4.2.1 (R Core Team, 2022). R is a language and environment for statistical computing (R Foundation for Statistical Computing, Vienna, Austria). The categorical variables were expressed as numbers (N) and percentages (%). Independent groups were analyzed using Pearson’s chi-square test (if expected frequencies > 5) and Fisher’s exact test (if expected frequencies < 5). Odds ratio (OR) calculations were used to evaluate 2 × 2 tables. The normality of the distribution of continuous variables was assessed using the Shapiro–Wilk test. Normally distributed continuous variables were presented as the mean (M) and standard deviation (SD). Data that were not normally distributed were presented as the median (Md) and interquartile range (Q1–Q3). Two independent, normally distributed samples were compared using Student’s independent samples *t*-test; otherwise, the Mann–Whitney test was applied. Pearson’s correlation coefficient (denoted *r*) was used to calculate the correlation between two normally distributed quantitative variables, and Spearman’s correlation coefficient (denoted *r_s_*) was used for non-normally distributed variables. The following classification was used to interpret correlation strength: <0.3 = weak, 0.31–0.69 = moderate, and >0.7 = strong. A 95% confidence interval (95% CI) was calculated to evaluate the precision of statistical estimates. For all statistical analyses, a *p*-value of <0.05 was considered statistically significant.

#### Ethical Considerations

This study was conducted in accordance with the Declaration of Helsinki and was approved by the Ethics Committee at Riga East University Hospital Ethics Board (No. 79/2018, 1 November 2018) and Rīga Stradiņš University Ethics Board (No. 6-3/120, 29 November 2018). The patients provided their written informed consent to participate in this study.

## 4. Results

### 4.1. General Characteristics

This study included a total of 84 participants: 42 patients with RRMS with a disease duration of less than 20 years and 42 volunteers in the control group without neurological dysfunction. In the MS group, 19 participants (45.2%) were female and 23 (54.8%) were male; in the control group, there were 25 (59.9%) females and 17 (40.5%) males. The average age in the MS group was 38.5 years [min 22; max 57], and in the control group, it was 34.0 years [min 22; max 62]. Both groups were divided by education level: higher or secondary education. In the MS group, 27 respondents (64.3%) had higher education compared to 23 (54.8%) in the control group. There was no statistically significant difference in demographics between the MS group and the control group.

In the MS group, the median duration of the illness was 7.00 years [Q1 5.00; Q3 9.00], and the median EDSS score was 2.5 [Q1 2.00; Q3 3.5]. Both groups were assessed using the SDMT, BVMT-R learning, and 9-HPT tests for both hands. Additionally, the pNfL level was measured in the MS group, with a median pNfL level of 6.40 pg/mL [Q1 4.40; Q3 10.9] (Table 1).

### 4.2. Cognitive Assessment Results

**Figure 1:**
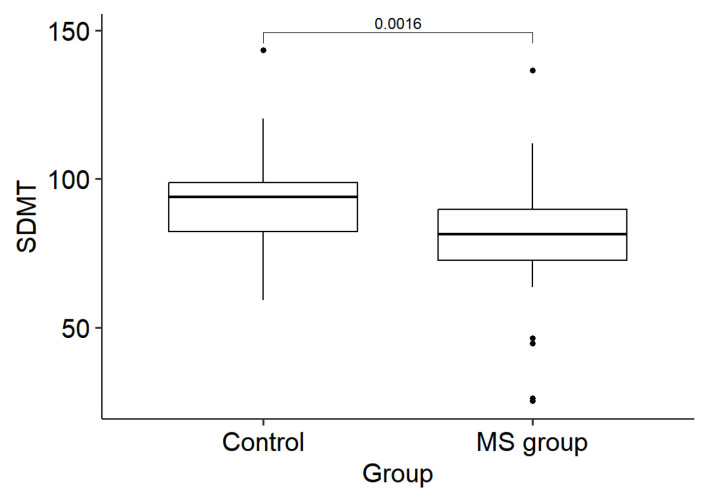
SDMT scores for the control and MS groups.

In the written SDMT, there was a significant difference between the two groups for cognitive processing speed (*p* = 0.002); the MS group obtained a median score of 81.3 [Q1 72.5; Q3 89.7], while the control group scored 94.0 [Q1 82.3; Q3 98.8] (*p* = 0.002), signifying a reduced processing speed in MS patients (Figure 1). The SDMT was compared in both groups based on education level (secondary or higher education). The significant difference between the MS group and the control group was maintained (*p* < 0.05), with individuals having higher education showing better performance (*p* < 0.05).

In Pearson’s correlation coefficient analysis, a negative, moderate, and statistically significant correlation was found between the EDSS and the SDMT (r = 0.56, *p* < 0.001). MS patients with higher disability levels had a poorer SDMT performance.

In the BVMT-R test for learning ability, there was a significant difference between the MS and control groups (*p* < 0.05). The MS group’s median score was 2.50 [Q1 2.00; Q3 3.00], and in the control group, the median score was 2.00 [Q1 1.00; Q3 4.00], suggesting that the capacity for cognitive improvement and learning ability was better in the control group. Education level did not affect the BVMT-R test results in either group, and there was no statistically significant difference between education level and learning ability (*p* = 0.91). In the MS group, learning was influenced by age, with a positive, weak, and statistically significant correlation (r = 0.26; *p* = 0.045). No significant difference was found between sexes in the MS group (*p* < 0.05). Pearson’s correlation coefficient analysis between the EDSS and learning (*p* = 0.42) and pNfL and learning (*p* = 0.60) did not establish a statistically significant correlation.

### 4.3. Fine Motor Skills Results

**Figure 2:**
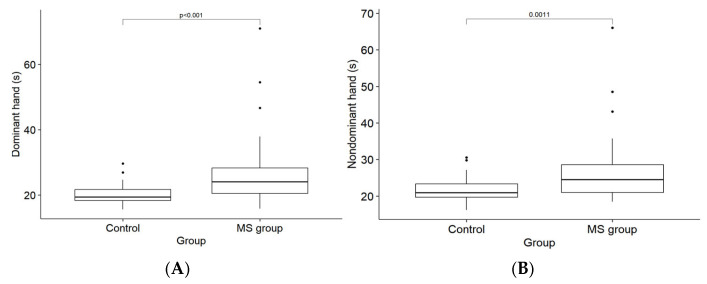
(**A**) Dominant hand reaction time (in seconds) for the control and MS groups. (**B**) Nondominant hand reaction time (in seconds) for the control and MS groups.

During the 9-HPT for the dominant hand, the respondents in the MS group took a median of 24.1 s [Q1 20.5; Q3 28.3], and for the nondominant hand, a median of 24.5 s [Q1 21.0; Q3 28.6]. The control group performed faster, with a median of 19.4 s [Q1 18.3; Q3 21.7] (Figure 2A) for the dominant hand and a median of 20.9 s [Q1 19.7; Q3 23.3] (Figure 2B) for the nondominant hand. The reaction times revealed a significant disparity between the MS patients and healthy controls (dominant hand *p* < 0.001; nondominant hand *p* < 0.05); the MS patients demonstrated slower reaction times, and their fine motor skills were affected.

**Figure 3:**
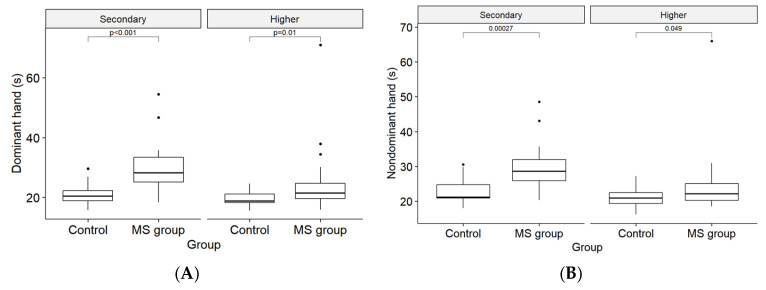
(**A**) Dominant hand reaction time (in seconds) by education level in the control and MS groups. (**B**) Nondominant hand reaction time (in seconds) by education level in the control and MS groups.

When stratified by education level for both the MS and control groups, it was determined that the observed difference was not associated with the level of education in the 9-HPT for either the dominant or nondominant hand (Figure 3A,B).

### 4.4. Neurofilament Light Chain Levels in Plasma

**Figure 4:**
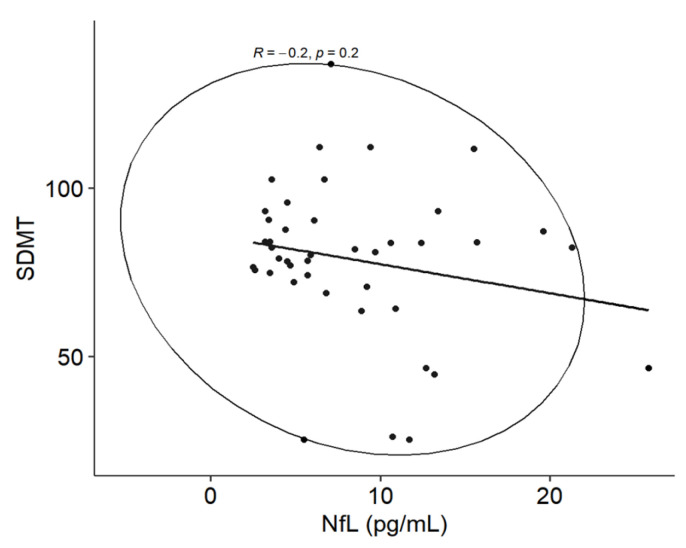
Correlation between pNfL and the SDMT in the MS group.

For the MS group, an analysis of the relationship between pNfL and the SDMT did not reveal a statistically significant correlation (*p* = 0.2); however, a potential visual relationship between pNfL and the SDMT was observed (Figure 4). Similarly, no statistically significant correlation was detected between pNfL and BVMT-R learning in the MS group (*p* = 0.6). Additionally, Pearson’s correlation coefficient analysis between the EDSS and pNfL did not indicate a statistically significant correlation (*p* = 0.2).

## 5. Discussion

The aim of this study was to investigate the correlation between functional status, cognitive function, and pNfL levels in RRMS patients. Assessing physical and cognitive functions in MS patients is important for monitoring disease progression and determining the effectiveness of therapy. The progression of the disease can occur only with the deterioration of cognitive status. There are many studies on this topic, and various biomarkers for disease progression are being investigated; the best biomarkers for disease diagnosis, prognostic, and therapeutic efficacy are being sought. It is important to find a combination of tests and biomarkers that would indicate the progression of the disease and could be used in daily practice to monitor the patients. In daily practice, it is important that the tests are quick and easy to apply. We chose tests that are fast, easy to apply, and have no language barrier and a biomarker that is easy to detect.

For the cognitive evaluation in MS patients, it is recommended to use the Brief International Cognitive Assessment for MS (BICAMS); the battery includes tests of mental processing speed and memory. The BICAMS includes the SDMT, the California verbal learning test, and the BVMT-R [20]. We used two of these—the SDMT and BVMT-R—to assess processing and motor speed, attention, visual scanning, visual–spatial memory, learning, and memory abilities. The SDMT is considered the gold standard for screening cognitive impairment in MS patients [21] and is recommended in MS trials as a tool for assessing cognitive processing speed [22]. Sandry et al. stated that the SDMT is a unique and sensitive tool that is capable of detecting multiple cognitive processes in MS patients, including memory, information processing speed, and lexical access speed. In addition, it is easy and quick to apply in daily practice, making it well suited for the demands of a busy daily life [21,23]. The results from the SDMT also indicate a significant disparity between healthy individuals and those with MS, corroborating the findings reported by Patel et al. in 2017 [24]. This underscores the effectiveness of the SDMT as an assessment tool for distinguishing between individuals with MS and those without the condition. Consistent with our study, Lopez-Gonora et al. [25] and Grothe et al. [26] also found that the SDMT scores were lower in the MS group compared to the control group. The SDMT being correlated with functional outcomes has been reported in other studies [27,28], although the data are conflicting, and cognitive function is independent of functional disability [29]. Goldman et al. also did not find a correlation between the EDSS and the SDMT; they stated that the SDMT can be used as a tool to detect other functions that are not mentioned in the EDSS [27]. In the present study, we found that a higher EDSS resulted in worse SDMT results, slower thinking, and slower performance speed in MS patients. The influence of education level on SDMT performance has also been mentioned in other studies, where patients with higher education levels had better SDMT results at baseline [30,31].

Furthermore, no significant correlation between sex and education was identified in the study conducted by Simioni et al. in 2008 [32]. Our own findings align with this, indicating that demographic factors, such as sex distribution and age, are not substantial contributors to the observed cognitive differences, except for education level with higher education, which showed better performance for the SDMT. However, Al-Falaki et al. found that patients with higher education had better results despite exhibiting mild cognitive dysfunction [33]. This suggests that the main source of these cognitive disparities may indeed be attributed to the presence of the disease itself.

The BVMT-R is used to assess visuospatial memory and recall. The BVMT-R results were significantly better in healthy controls than in the MS group [34,35]. However, it should be noted that the literature presents conflicting evidence on this matter. In 1984, Grant et al. [36] suggested that the extent of short-term memory impairment is directly related to the stage of the disease and the medications administered. Simioni et al. [32] also supported this notion in 2008 by identifying a significant correlation between decreased learning ability and MS relapses. In the early stages of the disease, no substantial impairment in learning and decision-making abilities was observed. There are studies showing that both age [28,37,38,39] and level of education affect performance on the BVMT-R [40], which could be related to the normal aging process, and with higher levels of education, people engage their brains more. In the present study, education level was not correlated with learning in either group, but in the MS group, an effect of age was observed; however, this could be due to the small sample size. Similarly, there are other studies that have not found correlations between age, education level, and BVMT-R performance [35,41]; therefore, age and education level are still questionable influencing factors for the BVMT-R performance results regarding visuospatial memory and learning.

The 9-HPT is the gold standard for evaluating arm and hand function. Fine motor and hand function are often affected in MS patients, although these impairments are not reflected in the EDSS. We found that individuals with MS demonstrated a significant delay in their dominant hand reaction times compared to the reaction times observed in the healthy control group. To elaborate, MS patients exhibited slower reaction times, and this difference was found to be statistically significant. Our findings, in the context of the study conducted by Feys et al. [42] on the 9-HPT as a manual dexterity performance measure, provide valuable insights into the manual dexterity impairments observed in MS patients. In the present research specifically, tasks involving both dominant and nondominant hands, as well as tasks requiring the coordination of both hands simultaneously, exhibited significantly slower reaction times in MS patients. Our results reinforce the validity of the 9-HPT as a sensitive tool for detecting impairments in fine motor skills among MS patients. Our findings not only support but also extend the conclusions drawn by Feys et al. [42]; while their study primarily focused on manual dexterity, the present research adds a temporal dimension by emphasizing the critical role of reaction times. The slower reaction times observed in MS patients indicate potential neurological disturbances that affect the speed and precision of hand movements, complementing the assertion by Feys et al. [42] of impaired manual dexterity in this population. Solaro et al. recommend being careful when using the 9-HPT in patients with an EDSS < 3.0 or an EDSS > 6.0 [43].

Nowadays, various biomarkers for MS are actively studied in relation to diagnostics, prognostics, monitoring, and the evaluation of therapy effectiveness such us NfL, glial fibrillary acidic protein, and other proteins. The most studied biomarker in recent years is NfL. However, recent studies have shown that combined inflammatory and neurodegenerative protein biomarkers reveal a stronger correlation with clinical and magnetic resonance disease progression than NfL alone [44,45]. NfL is a nonspecific biomarker of MS, but it is a promising biomarker of axonal damage [46,47], and higher levels of NfL might reflect the increased inflammatory activity of the disease [44]. Aktas et al. found no relationship between pNfL and the SDMT for cognitive function. They stated that pNfL cannot be used as a biomarker of cognitive function in patients with stable MS [48], although the data are conflicting because Mattioli et al. found a correlation between NfL and cognitive function and expressed the possibility of using NfL as a biological marker of cognitive dysfunction in MS patients. They found higher NfL levels in MS patients with more pronounced cognitive deficits [49]. We obtained a small trend between the SDMT and pNfL in the present study; however, this was not confirmed by the BVMT-R. Therefore, at present, we have obtained results similar to those of Aktas et al. [48]. Rademacher et al. reported an association between NfL levels and impaired information processing speed, suggesting that NfL is a promising biomarker for cognitive impairment in MS patients [50]. pNfL levels are higher in cognitive impairment in progressive neurodegenerative central nervous system diseases, according to Gotze et al. [51]. There are publications in which pNfL levels correlate with cognitive status [52] and functional status [46,52] in MS patients, but in the present study, we did not find this. This may be attributed to the small sample size, the low EDSS, and remission of the disease. The routine use of pNfL as a biomarker in daily practice has not yet been possible due to cut-offs in defining normal values and the level of pNfL. There are many factors that influence its use, such as age and weight [11].

Despite this study’s inclusion of cognitive and EDSS score evaluation and a potential blood biomarker, pNfL, it has several limitations. Firstly, the relatively small number of participants restricted the statistical analysis, resulting in some ambiguous findings. The small cohort included a demographically similar group of MS patients—young patients with a short course of disease duration and a relatively low EDSS. Secondly, we did not include therapies that affect the course of the disease and cognitive functions. 

## 6. Conclusions

In conclusion, statistically significant differences in cognitive functions were confirmed between the MS and control groups—the SDMT results showed a significant difference between the MS and control groups. They are related to fast information processing rather than memory and learning. As the disease progresses and the EDSS increases, the patient’s cognitive abilities deteriorate. Cognitive status and the EDSS score were not associated with pNfL levels. Taking into account our study data, in the future, a larger cohort will need to be collected in order to express more information about the relationship between pNfL and cognitive and physical impairment in MS patients.

## Figures and Tables

**Table 1:** Demographics of the MS and control groups.

	Control Group	MS Group
	N = 42	N = 42
Sex, N (%):		
Female	25 (59.5%)	19 (45.2%)
Male	17 (40.5%)	23 (54.8%)
Age, Md [Q1; Q3]	34.0 [26.2; 48.0]	38.5 [30.0; 46.8]
Education, N (%):		
Higher	23 (54.8%)	27 (64.3%)
Secondary	19 (45.2%)	15 (35.7%)
Duration of MS, years Md [Q1; Q3]		7.00 [5.00; 9.00]
EDSS, Md [Q1; Q3]		2.5 [2.00; 3.5]
pNfL, pg/mL Md [Q1; Q3]		6.40 [4.40; 10.9]

## Data Availability

The data are available upon request due to ethical restrictions. Requests to access datasets should be directed to elinapolunosika@gmail.com. The data are not publicly available and are stored in the patient medical record repository at Riga East University Hospital.
